# Obesity and abnormal glucose tolerance in offspring of diabetic mothers: A systematic review and meta-analysis

**DOI:** 10.1371/journal.pone.0190676

**Published:** 2018-01-12

**Authors:** Maki Kawasaki, Naoko Arata, Celine Miyazaki, Rintaro Mori, Toru Kikuchi, Yoshihiro Ogawa, Erika Ota

**Affiliations:** 1 Department of Health Policy, National Center for Child Health and Development, Tokyo, Japan; 2 Department of Molecular Endocrinology and Metabolism, Graduate School of Medical and Dental Sciences, Tokyo Medical and Dental University, Tokyo, Japan; 3 Division of Maternal Medicine, Center for Maternal-Fetal, Neonatal and Reproductive Medicine, National Center for Child Health and Development, Tokyo, Japan; 4 Department of Pediatrics, Saitama Medical University Hospital, Saitama, Japan; 5 Department of Medicine and Bioregulatory Science, Graduate School of Medical Sciences, Kyushu University, Fukuoka, Japan; 6 St. Luke’s International University, Graduate School of Nursing, Global Health Nursing, Tokyo, Japan; Florida International University Herbert Wertheim College of Medicine, UNITED STATES

## Abstract

**Background:**

Rising prevalence of childhood obesity and type 2 diabetes mellitus (T2DM) is an emerging public health issue.

**Objectives:**

To investigate the association of maternal hyperglycemia exposure during pregnancy with obesity and abnormal glucose tolerance in offspring, and the age at occurrence.

**Methods:**

We searched MEDLINE and EMBASE for observational studies on obesity and diabetes in offspring of diabetic mothers (gestational diabetes mellitus (GDM), type 1 diabetes mellitus (T1DM) and T2DM), and those on non-diabetic mothers. We performed fixed effect meta-analysis for all studies except when heterogeneity was detected. The quality of studies was evaluated using the Risk of Bias Assessment Tool for Nonrandomized Studies (RoBANS)

**Results:**

Twenty observational studies were included involving a total of 26,509 children. Offspring of GDM mother had higher BMI z-score in childhood (pooled MD: 0.14, 95%CI: 0.04–0.24, seven studies, 21,691children, low quality of evidence). Offspring of T1DM mothers had higher BMI z-score from prepubertal to adolescent (pooled MD: 0.35, 95% CI: 0.13–0.58, three studies, 844 children, low quality of evidence) compared with control. After adjustment for maternal pre-pregnancy BMI, this association remained in offspring of T1DM, but disappeared in those of GDM mothers. Offspring of GDM mother had higher 2-hour plasma glucose from prepubertal to early adulthood (pooled MD: 0.43 mmol/L, 95% CI: 0.18–0.69, five studies, 890 children), while those of T1DM mothers had higher rate of T2DM in 2–5 years old to early adulthood (pooled odds ratio [OR], 6.10: 95% CI: 1.23–30.37, two studies, 448 children, very low quality of evidence) compared with control. As there was only one study with offspring of T2DM mothers, evidence is sparse.

**Limitations:**

Only observational studies were included, with a few adequately adjusted for covariables.

**Conclusions:**

Exposure to maternal hyperglycemia was associated with offspring obesity and abnormal glucose tolerance especially in offspring of T1DM mothers, but the evidence relies on observational studies with low quality of evidence only.

## Introduction

Prevalence of type 2 diabetes mellitus (T2DM) has increased globally, impacting on health and economies worldwide [1]. In particular, the increased prevalence of T2DM in children, adolescents and young adults [2], combined with increased childhood obesity, is a serious public health concern [1]. In 2014, 42 million children (6.1%) under 5 years old were overweight or obese worldwide, up from 31 million (4.8%) in 1990 [3]. It was predicted that if current trends continue, the number of children under 5 years old who are overweight or obese would rise to 70 million by 2025 [3]. Notably, overweight and obese children are likely to be obese as adults, and will have non-communicable diseases (NCDs) such as T2DM at a younger age [4].

Fuel-mediated teratogenesis is a well-known hypothesis regarding intrauterine exposure to maternal diabetes [5]. Exposure to hyperglycemia in utero is believed to be associated with offspring obesity and impaired glucose tolerance [5], which is one of the most important viewpoints in preventing sharp rise in obesity and type 2 diabetes in the near future.

To date, two systematic reviews have shown the association between maternal diabetes and childhood obesity [6, 7]. In both reviews, maternal hyperglycemia was shown to be a risk factor for obesity or overweight in offspring, but the association was attenuated or no longer apparent after adjusting for covariates, especially maternal BMI [6, 7]. To clarify this association, it is necessary to consider the differences in the timing and degree of hyperglycemic intrauterine exposure, the difference in the genetic predisposition of obesity and T2DM, and the difference in the evaluation age of offspring. At present, there is no systematic review on the association between maternal hyperglycemia and childhood abnormal glucose tolerance.

Therefore, we aimed to systematically review current findings on children with obesity and glucose metabolism born to diabetic mothers to clarify whether intrauterine hyperglycemia exposure increases the risk of obesity and abnormal glucose tolerance of offspring according to the type of maternal diabetes. We also sought to clarify at which age the impact of intrauterine maternal diabetes on child obesity or abnormal glucose tolerance emerged.

## Methods

### Data sources and searches

Reporting procedures for this systematic review were consistent with the Meta-analysis of Observational Studies in Epidemiology (MOOSE) reporting guidelines [8]. An information specialist conducted a comprehensive literature search of EMBASE and MEDLINE for reports on obesity and diabetes among offspring born to diabetic mothers published between January 1946 and December 2016. Then, we conducted hand searching and checked the references lists of the retrieved articles. We also searched all abstracts published by the American Diabetes Association and the European Association for the Study of Diabetes for their annual meetings in the last five years for inclusion. No language restrictions were applied. The detailed search strategy is shown in S1 Table.

### Study selection

We included studies that evaluated the association between intrauterine exposure to maternal hyperglycemia and offspring obesity and diabetes.

The inclusion criteria are as follows:

1. Exposures to maternal diabetes, including GDM, pre-pregnancy type 1 diabetes mellitus (T1DM) and T2DM. Studies with unclear diabetes status (e.g., only ‘pre-existing diabetes’) and with no information on whether the mothers had diabetes before or after their pregnancies were excluded;
2. Non-diabetic control group;
3. Offspring were from a singleton pregnancy;
4. Offspring were over 2 years old;
5. One of the following outcomes was described:

Primary outcomes: the prevalence of obesity or overweight, BMI z-score, the prevalence of DM, secondary outcomes: fasting plasma glucose and 2-hour plasma glucose, and the prevalence of abnormal glucose tolerance (DM, impaired fasting glucose (IFG), impaired glucose tolerance (IGT)).

The definition of obesity or overweight is a BMI of >85^th^ or >95^th^ percentile for age and sex. The 2-hour plasma glucose (2hPG) and glucose tolerance were determined by a 2-hour oral glucose tolerance test.

Study designs such as randomized controlled trials (RCTs) and prospective or retrospective cohort studies were considered for inclusion. If offspring outcomes were described at multiple ages, we used findings for the longest duration of follow-up. The Pima Indian cohort was excluded as they have a genetically high incidence of obesity and T2DM. Reports were limited to human studies without any language restriction.

### Data extraction and quality assessment

Eligible titles and abstracts were screened independently by two researchers (MK and CM). After screening, full-text articles of relevant studies were obtained. Any disagreements were resolved through discussion or consulting a third reviewer (EO).

Data were independently extracted by two researchers (MK and CM) from included reports using a data extraction form. Extracted data included the following: first author’s name, year of publication, study design, study duration, number of participants, maternal characteristics (pre-pregnancy BMI), exposure (maternal diabetes status and evidence of diagnosis), offspring characteristics (age, sex and ethnicity), outcome and covariates. We contacted authors directly for additional data relating to missing information. We divided age of offspring into four categories: 2 to 6 years old, 7 to 9 years old, 10 to 15 years old, 16 to 19 years, and over 20 years old.

The quality of studies was evaluated using the Risk of Bias Assessment Tool for Nonrandomized Studies (RoBANS) [9]. The RoBANS tool was developed for assessing the risk of bias of non-randomized studies, and comprised six domains: selection of participants, confounding variables, measurement of exposure, blinding of outcomes, incomplete outcome data and selective outcome reporting. The risk of bias for each domain was classified as low risk, high risk and unclear risk.

### Data synthesis and analysis

Data from included studies were pooled and meta-analysis was performed. For dichotomous data, odds ratios (ORs) were calculated using a fixed-effects model. When heterogeneity was detected, a random-effects model was used. For continuous data, mean differences (MDs) with 95% confidence interval (CI) were used. Heterogeneity between studies was evaluated using the I^2^ statistics. We regarded heterogeneity as substantial if I^2^ was greater than 75%. All statistical analyses used 95% CI and a p-value with a cut-off point of 0.05. All statistical analyses were performed using Review Manager version 5 software (RevMan 5.3; The Cochrane Collaboration, Oxford, UK).

If the domain of participant selection was determined by RoBANS to be high risk, we conducted sensitivity analysis and excluded the research from the meta-analysis [10]. We extracted data by age of offspring. If there was no significant subgroup difference, we synthesized the data. If there was a significant subgroup difference, we interpreted the data according to age.

### Evidence grading

#### GRADE

We evaluated the quality of evidence with the Grading of Recommendations Assessment, Development and Evaluation (GRADE) approach using GRADEpro GDT available at http://guidelinedevelopment.org.

Quality ratings were made for the BMI z-scores, as well as the ORs for obesity or overweight, DM and the abnormal glucose tolerance of mothers with GDM and controls, and mothers with T1DM.

## Results

### Literature search

**Fig 1:**
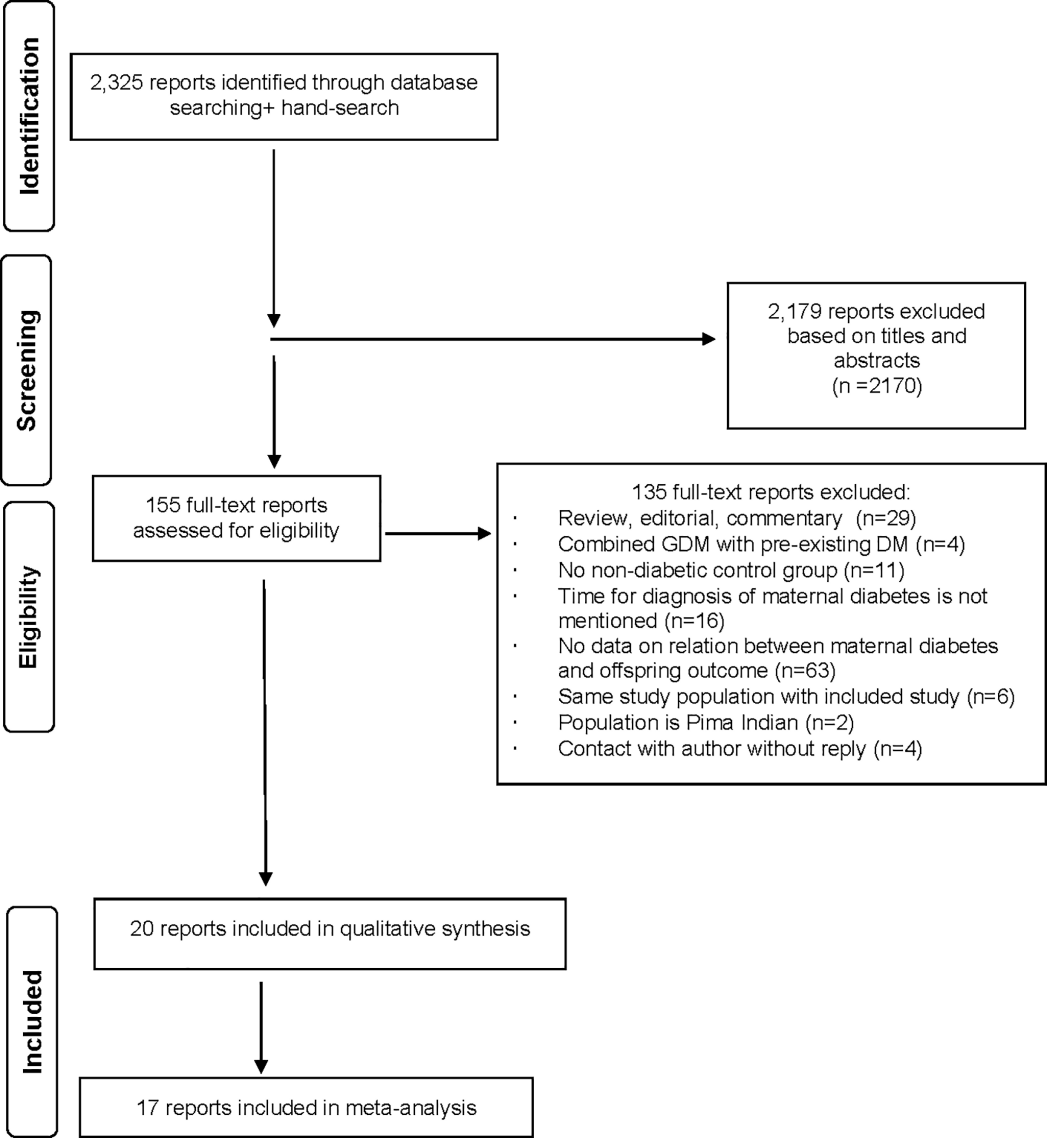
PRISMA flow diagram of search and study selection.

We identified 2,325 reports through our database searches (Fig 1). Of these, 2,170 were not relevant and were excluded based on the title and abstract, leaving 155 articles for full-text evaluation. Of the 155 studies, 132 were excluded due to outcomes of interest not reported, control group not included and timing of maternal diagnosis not reported. We translated one study and contacted the authors of six studies.

Finally, we selected 20 studies to be included in the current systematic review. Of these, 14 studies included offspring of mothers with GDM, eight studies focused on offspring of mothers with T1DM and one study included offspring of mothers with T2DM.

### Study characteristics

The characteristics of the 20 included studies [11–30] are summarized in Table 1.

**Table 1:** Characteristics of included studies.

**Offspring·of·mothers·with·gestational·diabetes·mellitus¤**									
**Autdor, year¤**	**GDM·criteria,** **Treatment (Tx)¤**	**Offspring·characteristics¤**	**Outcome¤**	**GDM·group¤**		**Control·group¤**		**P·value¤**	**Adjustment¤**
**Prospective·study¤**									
**Boerschmann, 2010¤**	OGTT* two·of·three:·fasting≧90mg/dl,1h·180mg/dl, 2h≧155mg/dl Tx:NA¤	Age = 11·years Germany Race/ethnicity: NA Sex: NA¤	BMI>90th percentile¤	N = 77¤	n = 24¤	N = 148¤	n = 23¤	NA**	Not·done¤
**Catalano, 2009¤**	National·Diabetes·Group·criteria Tx:diet(+insulin)¤	Age = 6.1–11.9 years USA Race/ethnicity:White,African American,Hispanic Sex: NA¤	BMI·z-score	N = 37	0.9±1.4	N = 52	0.31±1.16	0.03	Not·done
			FPG**** ¤	N = 23¤	4.9±0.3¤	N = 26¤	4.8±0.2¤	0.33¤	Not·done¤
**Davis, 2013¤**	Self-report Tx:NA¤	Age = 8–13·years USA Race/ethnicity: Hispanic Sex: female,GDM: 49%, control:42%¤	BMI·z-score	N = 47¤	2.2±0.4	N = 163¤	2.1±0.4	NS	Not·done
			FPG****		4.99±0.38		4.94±0.34	NS	Not·done
			2hPG***** ¤		7.19±0.96¤		7.03±1.02¤	NS¤	Not·done¤
**Holder, 2014¤**	Responded·to a·validated questionnaire Tx:NA¤	Age = 4–20·years (Mean age = GDM:15, Control:15) USA Race/ethnicity: NA Sex: NA¤	BMI·z-score	N = 45¤	2.37±0.54	N = 219	2.26±0.58	0.27	Not·done
			FPG****		5.27±0.5	N = 210¤	5.11±0.5	0.01	Not·done
			2hPG*****¤		7±1.44¤		6.33±1.11¤	0.005¤	Not·done¤
**Pirkola, 2010¤**	National·guidelines·in·Finland Tx:Diet,SMBG,(+insulin)¤	Age = 16·years Finland Race/ethnicity: NA Sex: NA¤	BMI>85th·percentile¤	N = 84¤	n = 18¤	N = 661¤	n = 113¤	NA¤	Not·done¤
**Tam, 2010¤**	WHO·criteria·1999 Tx:NA¤	Age = 15·years Hong Kong Race/ethnicity:Chinese Sex: NA¤	BMI≧85th· percentile	N = 63	N = 63	N = 101	n = 26	0.51	Not·done
			DM	N = 42¤	n = 1	N = 87¤	n = 0	0.77	Not·done
			FPG****		4.6±0.3		4.7±0.3	0.51	Not·done¶
			2hPG*****¤		6±1.5¤		5.6±1.4¤	0.16¤	Not·done¤
**Whitaker, 1998¤**	Carpenter·and·Cousta·criteria Tx:diet¤	Age = 8–10·years USA Race/ethnicity: NA Sex: NA¤	BMI·z-score	N = 58¤	0.39±0.94	N = 257¤	0.45±0.93	0.4	Not·done¶
			BMI>85th·percentile¤		n = 11¤		n = 62¤	0.4¤	Not·done¤
**Wright, 2009¤**	Carpenter·and Cousta·criteria Tx:diet,·exercise,(+insulin)¤	Age = 3·years USA Race/ethnicity:White,Black, Hispanic,Other Sex: female,GDM:45%, control:49%¤	BMI·z-score	N = 51¤	0.47±1.2	N = 1053¤	0.44±1.02	0.68	-0.08±0.15(p = 0.61)
			BMI>85th·percentile¤		n = 9¤		n = 169¤	0.52¤	Not·done¤
**Retrospective·study¤**									
**Buzinaro,2008¤**	medical·record·and·questionnaire Tx:diet(+insulin)¤	Age = <17·years (Mean age = GDM:15,control:12) Brazil Race/ethnicity: NA Sex: NA¤	BMI>85th·percentile	N = 23¤	n = 12	N = 27¤	n = 4	NA**	Not·done¤
			FPG****¤		5.17±0.34¤		5±0.39¤	NA**	Not·done¤
**Clausen,2008¤**	OGTT*, OGTT·at ·east·two·of·seven·glucose·values ·exceeded·the·mean·3SD·values·for·a·reference·group·of·normal-weight·non-pregnant·women· without·a·family·history·of·diabetes Tx:NA¤	Age = 18–27·years Denmark Race/ethnicity: NA Sex: female, GDM: 46%, control:51%¤	DM	N = 168¤	n = 7	N = 128¤	n = 1	0.77	Not·done
			IGT		n = 19		n = 3		
			IFG		n = 10		n = 1		
			FPG****		5.5±0.9		5.1±0.4	<0.001	Not·done
			2hPG*****¤		5.9±2.1¤		5.3±1.3¤	0.005¤	Not·done¤
**Gillman,2003¤**	Self-report Tx:NA¤	Age = 9–14·years USA Race/ethnicity: NA Sex: female, GDM: 50%, control:54%¤	BMI·z-score	N = 463¤	0.33±1.01	N = 14416¤	0.15±0.12	<0.001	Not·done
			BMI>95th·percentile¤		n = 45¤		n = 958¤	NA**¤	OR·1.2(0.8–1.7) ¤
**Page,2014¤**	NA¤	Age = 5–16·years USA Race/ethnicity:Mexican·American Sex: female, GDM: 52%,control:27%¤	BMI·z-score¤	N = 25¤	0.95±0.2¤	N = 37¤	0.25±0.2¤	0.02¤	Not·done¤
**Patel, 2012¤**	By questionnaire Tx:NA¤	Age = 15.5·years UK Race/ethnicity: NA Sex: NA¤	BMI z-score	N = 27¤	0.37±1.11	N = 4834¤	-0.22±0.97	NA**¤	-0.15±0.19¤
			FPG****¤		5.4±0.47¤		5.21±0.38¤	NA**¤	Not·done¤
**Pham,2013¤**	National·Diabetes·Group·criteria,·switched·to·the·Carpenter·and·Coustan·criteria, Tx:participated·in·an·educational· class·focusing·on·diet·modification,postprandial·exercise,blood ·glucose·monitoring,appropriate ·weight·gain.	Age = 2–4·years USA Race/ethnicity: South·Asian, Asian, Black, Latina, White, Other Sex: NA¤	BMI>85th·percentile¤	N = 255¤	n = 61¤	N = 1838¤	n = 432¤	NA**¤	Not·done¤
**Offspring·of·mothers·with·type 1·diabetes·mellitus¤**									
**Author, year¤**	**T1DM·criteria,** **Treatment¤**	**Offspring·characteristics¤**	**Outcome¤**		**T1DM·group¤**		**Control·group¤**	**P·value¤**	**Adjustment¤¤**
**Prospective·study¤**									
**Boerschmann,** **2010¤**	WHO·criteria. insulin¤	Age = 11·years Germany Race/ethnicity: NA Sex: NA¤	BMI>90th·percentile	N = 284¤	n = 45¤	N = 148¤	n = 23¤	NA**¤	Not·done¤
**Buinauskiene,** **2004¤**	WHO·criteria.¤	Age = 2–5·years Lithuania Race/ethnicity: NA Sex: female T1DM 45%, control:45%¤	DM¤	N = 51¤	n = 1¤	N = 109¤	n = 1¤	NS***¤	Not·done¤
**Lindsay, 2010¤**	medical·records, insulin¤	Age = 7.4·years UK,Scotland Race/ethnicity: NA Sex: NA¤	BMI·z-score	N = 100	0.69±1.2	N = 45	0.28±0.7	0.22	TIDM:0.67±0.11 Control:0.33±0.16 (p = 0.08)
			BMI>90th·percentile		n = 22		n = 0	0.001	Not·done
			FPG****	N = 53	4.5±0.3	N = 19	4.5±0.4	NA**	Not·done
			2hPG*****¤	N = 34¤	5.1±1.3¤	N = 12¤	5.7±0.8¤	NA**¤	Not·done¤
**Rodrigues, 1998¤**	NS¤	Age = 18–27·years Denmark Race/ethnicity: NA Sex: NA¤	BMI>95th·percentile¤	N = 17¤	n = 7¤	N = 18¤	n = 0¤	NA**¤	Not·done¤
**Vlachov, 2015¤**	medical·records¤	Age = 13–19.8·years Denmark Race/ethnicity: NA Sex: female, T1DM: 59%, control: 60%¤	BMI·z-score	N = 278¤	0.69±1.27	N = 303¤	0.24±1.14	<0.001	TIDM·0.44·higher ·than·control(p<0.001)
			FPG*****		5.4±0.4		5.3±0.4	0.021	TIDM·0.1·higher ·than·control(p = 0.008)
			2hPG****¤		6.4±1.3¤		6.1±1.2¤	0.009¤	TIDM· 0.2 ·higher ·than·control(p = 0.136)¤
**Retrospective·study¤**									
**Clausen, 2008¤**	fulfilled·three·criteria: onset·of ·diabetes·at·age≦40·years, classical·history, and·insulin·treatment·starting≦6·months ·after· diagnosis¤	Age = 5.9–9·years USA Race/ethnicity: NA Sex: female, T1DM 46%,control:51%¤	IGT	N = 160¤	n = 8	N = 128¤	n = 3	NS***	Not·done
			IFG		n = 6		n = 1	NS***	Not·done
			DM		n = 10 (T1·7,T2·3)		n = 1	NS***	Not·done
			FPG****		5.2±0.5		5.1±0.4	NS***	Not·done
			2hPG*****¤		5.8±1.6		5.3±1.3	NS***	Not·done¤
**Hunter, 2004¤**	Onset·before·30·years·and·one·and·more·of·the·following: autoantibody·positive (GAD, insulin-associated·protein·2,or ·islet cells), ketoacidosis·at presentation,·normal ·BMI·at·diagnosis·no·first-degree ·relative·with· type 2· diabetes, and ·commencement· of· insulin ·therapy· at· diagnosis, insulin¤	Age = 5–10·years New Zealand Race/ethnicity: NA Sex: female, T1DM: 24%, control: 47%¤	BMI·z-score	N = 17¤	0.7±0.6	N = 15¤	-0.2±0.6	0.22	Not·done
			FPG****¤		4.9±0.1¤		4.9±0.1¤	NS***¤	Not·done¤
**Manderson, 2002¤**	medical record database and standard questionnaire, insulin¤	Age = 5–11·years UK Race/ethnicity: NA Sex: female T1DM: 57%, control: 41%¤	BMI·z-score	N = 61¤	0.59±1.35	N = 57¤	0.6±1.21	0.96	Not done
			FPG****¤		4.35±0.32¤		4.44±0.28¤	0.16¤	Not done¤
**Offspring·of·mothers·with·type 2·diabetes·mellitus¤**									
**Author, year**	**T2DM criteria,** **Treatment¤**	**Offspring·characteristics¤**	**Outcome¤**	**T2DM·group¤**	**Control·group¤**	**P·value¤**	**Adjustment¤**
**Retrospective·study¤**									
**Hunter, 2004¤**	BMI>30kg/m^2^·at·diagnosis·had ·one· or· more ·of ·the· following: no·insulin· therapy requirement·non-ketoacidosis ·prone, and· the· presence· of ·acanthosis· nigricans.¤	Age = 5–10·years New Zealand Race/ethnicity: NA Sex: female, T2DM: 20%, control: 47%¤	BMI·z-score	N = 17¤	3.2±0.7	N = 15¤	-0.2±0.6	<0.001	Not·done¤
			FPG****¤		5±0.1¤		4.9±0.1¤	NS¤	Not·done¤

*OGTT, oral glucose tolerance test;

**NA, not applicable;

***NS,not significant,

****FPG, fasting plasma glucose;

***** 2hPG, two hours plasma glucose

#### Offspring of mothers with gestational diabetes mellitus

Fourteen studies of offspring of GDM mothers and controls were included in this review, involving a total of 25,336 children [11–17, 20–22, 24, 26, 27, 29]. Eight studies were prospective cohort [11, 13, 15–17, 20, 26, 29] and six were retrospective cohort [12, 14, 21, 22, 24, 27]. Eight studies were conducted in the USA [11, 13–17, 24, 27], one each in Germany [26], Finland [29], Hong Kong [20], Brazil [21], Denmark [22] and the UK [12]. Ethnicities of offspring were White [11, 13, 24], Hispanic [11, 13, 16], African American [13], Mexican American [14], Black [11, 24], South Asian [24] and Chinese [20]. The age of offspring ranged from 3 to 27 years old. The sex of offspring was available in five studies [11, 14, 16, 22, 27] and the proportion of boys and girls was about the same. The GDM diagnostic criteria were available in all studies, except for one [14].Two studies [11, 15] used Carpenter-Coustan criteria, two studies [13, 24] used National Diabetes Data Group criteria, one study [20] used WHO criteria, three studies [22, 26, 29] used other criteria and five studies [12, 16, 17, 21, 27] were based on self-report. GDM treatments were included in five studies [11, 13, 15, 21, 29].

#### Offspring of mothers with type 1 diabetes mellitus

Eight studies of offspring of T1DM mothers and controls were included in this review, involving a total of 4,957 children [18, 19, 22, 23, 25, 26, 28, 30]. Five studies were prospective cohort [23, 25, 26, 28, 30], and three were retrospective cohort [18, 19, 22]. Two studies were conducted in the UK [19, 28] [19, 28]and Denmark[23, 30], one each in Lithuania [25], Germany [26], the USA [22] and New Zealand [18]. The age of offspring ranged from 3 to 27 years old.

#### Offspring of mothers with type 2 diabetes mellitus

Only one study of offspring of T2DM mothers and controls was included in this review [18]. The study, involving 32 children, was a retrospective cohort study conducted in New Zealand and the age of offspring ranged from 5 to 10 years old.

### Quality evaluation

#### Risk of bias

The results of risk of bias assessment using RoBANs are summarized in S2 Fig. As for selection of participants, 55% studies were a low risk of bias, 15% at high risk and the risk was unclear for 30%. For incomplete outcome data, 30% studies were a low risk of bias, 30% at high risk and the risk was unclear for 40%. For selective outcome reporting, 5% studies were a low risk of bias, 25% at high risk and the risk was unclear risk for 75%.

### Association

#### Offspring of mothers with gestational diabetes mellitus

**Obesity and overweight**

**Fig 2:**
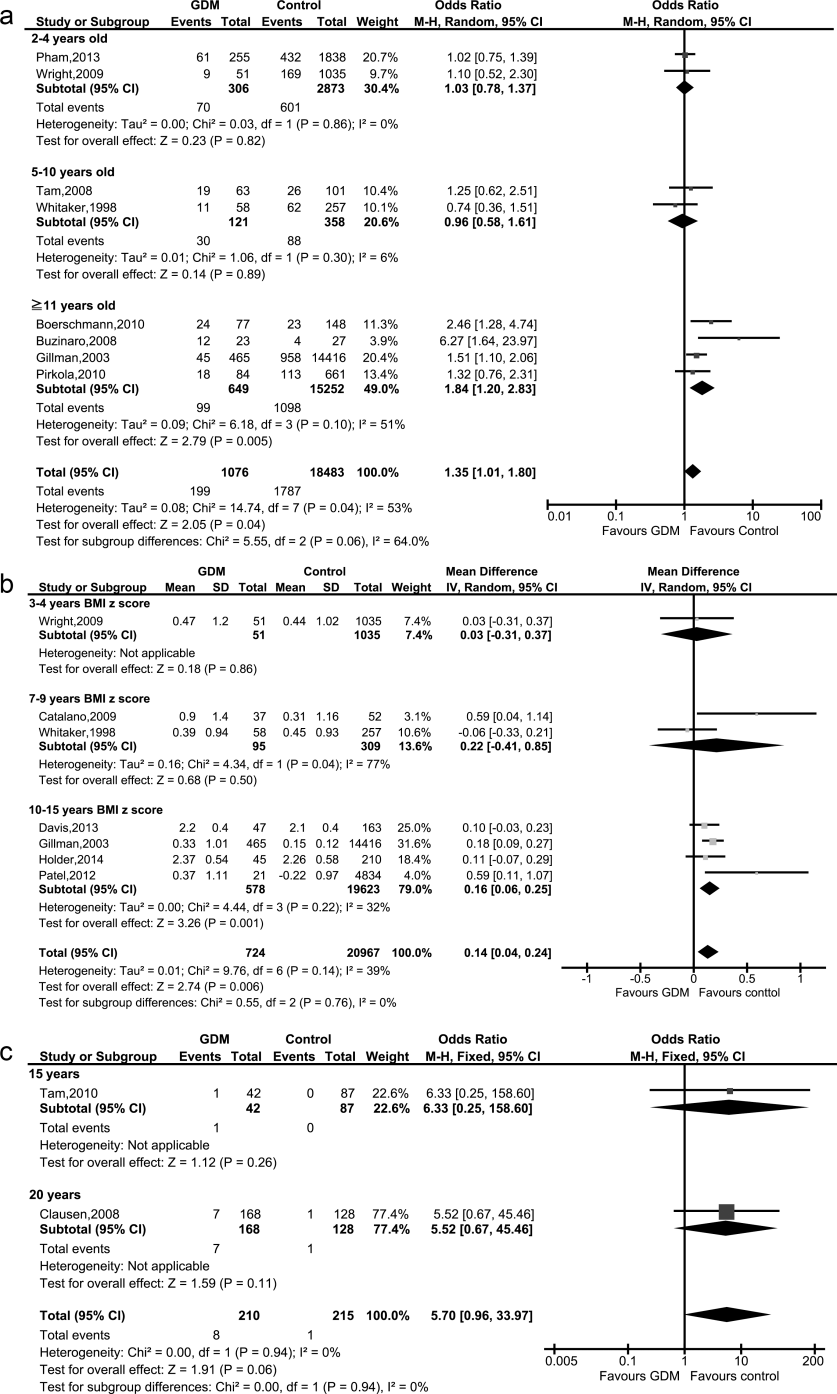
Forest plots of metabolic parameters in offspring of mothers with gestational diabetes mellitus and controls: (a), obesity or overweight, (b) BMI z-scores, (c) diabetes.

Eight studies of GDM mothers’ offspring and controls were included in this review, involving a total of 19,559 children [11, 15, 20, 21, 24, 26, 27, 29]. A forest plot of the crude odds ratios of obesity or overweight of mothers with GDM and controls is shown in Fig 2A.

The rate of childhood obesity or overweight was statistically significantly higher in the offspring of GDM mothers (p value = 0.04, pooled OR: 1.35; 95% CI: 1.01–1.80; I^2^ = 53%; eight studies, 19,559 children). Quality of evidence was judged as ‘low’ (S2 Table).

Only one study [27] was adjusted for covariates including maternal pre-pregnancy BMI. Therefore, we could not conduct an adjusted OR. Unadjusted OR for overweight in adolescent was 1.4 (95% CI: 1.0–1.9) and adjusted OR was 1.2 (95% CI: 0.8–1.7).

**BMI z-scores**

Eight studies of GDM mothers’ offspring and controls were included in this review, involving a total of 21,753 children [11–17, 27]. A forest plot of BMI z-scores in the offspring of GDM mothers and controls is shown in Fig 2B.

Unadjusted BMI z-scores in the offspring of GDM mothers was significantly (p value = 0.006) higher than in the controls (pooled MD: 0.14; 95%CI: 0.04–0.24; I^2^ = 39%; seven studies, 21,691 children) (Fig 2B). Quality of evidence was judged as low’ (S2 Table). In a sensitivity analysis, overall unadjusted BMI z-scores of the offspring of GDM mothers including Page et al. was significantly (p value = 0.03) higher than the controls (pooled MD: 0.26; 95%CI: 0.03–0.49; I^2^ = 92%; eight studies, 21,753 children) (S3A Fig), we excluded Page et al. [14] because the ‘selection of participants’ domain was high risk [31] and reported as main results. Heterogeneity decreased from 92% to 39% but significance did not change after sensitivity analysis (S3A Fig). Only two studies [11, 12] were adjusted for covariates including maternal pre-pregnancy BMI. Using the adjusted data, the association was no longer significant (pooled MD: -0.11; 95% CI: -0.33–0.12; two studies, 5,941 children).

**Diabetes**

Two studies of GDM mothers’ offspring and controls were included in this review, involving a total of 425 children [20, 22]. There was no significant difference in the rate of childhood diabetes between GDM and controls (pooled OR: 5.70; 95% CI: 0.96–33.97; I^2^ = 0%; two studies, 425 children) (Fig 2C)

From the viewpoint of abnormal glucose tolerance (total T2DM, IGT and IFG), no meta-analysis was conducted because of subgroup differences were detected (p value<0.05). There was no significant difference in children aged 15 years old (OR: 1.17; 95% CI: 0.37–3.74; 129 children). However, a significantly higher rate of abnormal glucose tolerance (p = 0.0001) was observed in offspring with GDM aged 20 years old (OR: 6.71; 95% CI: 2.55–17.65; 296 children, one study) (Table 2, S1A Fig). Quality of the evidence was judged as ‘very low’ and was downgraded due to the serious extent of loss to follow-up (S2 Table 2).

**Table 2:** Abnormal glucose tolerance and plasma glucose in offspring of mothers with gestational diabetes mellitus, type1 diabetes and controls.

**Offspring·of·mothers·with·gestational·diabetes·mellitus¤**					
**Outcome·or·subgroup·title¤**	**No.·of ·studies¤**	**No.·of·children¤**	**No.·of· control·children¤**	**Statistical·methods¤**	**Effect·size¤**
Abnormal·glucose·tolerance					
15· years	1	42	87	Odds·ratio (M-H,Fixed, 95%CI)	1.17 [0.37, 3.74]
20· years¤	1¤	168¤	128¤	Odds·ratio (M-H,Fixed, 95%CI)¤	6.71 [2.55, 17.65]¤
Fasting·plasma·glucose					
7–10·years	2	70	189	Std.·Mean·Difference (Ⅳ,Random,95%CI)	0.07 [-0.02, 0.16]
15·years	4	137	5158	Std.·Mean·Difference (Ⅳ,Random,95%CI)	0.09 [-0.07, 0.26]
20·years¤	1¤	168¤	128¤	Std.·Mean·Difference (Ⅳ,Random,95%CI)¤	0.40 [0.25,0.55]¤
2h·plasma·glucose					
7 ·to·20·years¤	4¤	302¤	588¤	Std. Mean Difference (Ⅳ, Random, 95%CI)¤	0.43 [0.18, 0.69]¤
**Offspring of mothers with type 1 diabetes mellitus¤**					
**Outcome·or·subgroup·title¤**	**No.·of· studies¤**	**No· of·children¤**	**No.·of·control·children¤**	**Statistical·methods¤**	**Effect·size¤**
Abnormal·glucose·tolerance					
2–5·years,20·years¤	2¤	211¤	237¤	Odds·ratio (M-H, Fixed, 95%CI)¤	3.48 [1.87, 6.49]¤
Fasting·plasma·glucose					
7–10·years	2	114	76	Std.·Mean·Difference (Ⅳ,Random,95%CI)	-0.07 [-0.16, 0.03]
15·years	1	278	303	Std.·Mean·Difference (Ⅳ,Random,95%CI)	0.10 [0.03, 0.17]
20·years¤	1¤	160¤	128¤	Std· Mean·Difference (Ⅳ, Random,95%CI)¤	0.10 [-0.00,0.20]¤
2h·plasma·glucose					
7–10·years	1	34	12	Std.·Mean·Difference (Ⅳ,Random,95%CI)	-0.60 [-1.23, 0.03]
15·years	1	278	303	Std.·Mean·Difference (Ⅳ, Random,95%CI)	0.30 [0.10, 0.50]
20·years¤	1¤	160¤	128¤	Std.·Mean·Difference (Ⅳ,Random,95%CI)¤	0.50 [0.17,0.83]¤

**Plasma glucose**

Seven studies of GDM mothers’ offspring and controls were included in this review, involving a total of 5850 children for fasting plasma glucose (FPG) and 890 children for 2hPG [12, 13, 16, 17, 20–22].

For FPG, no meta-analysis not conducted because of subgroup differences were detected. No significant difference was found between GDM and controls in 7- to 10-year-olds (pooled MD: 0.07 mmol/L; 95% CI: -0.02–0.16; I^2^ = 0%; two studies, 259 children) and in 15-year-olds (pooled MD: 0.09 mmol/L:, 95% CI: -0.07–0.26; I^2^ = 76%; four studies, 5,295 children). However, significantly higher FPG (p<0.00001) was found in the offspring of GDM mothers aged 20 years old (MD: 0.40 mmol/L; 95% CI: 0.25–0.55; 296 children) (Table 2, S1B Fig).

In the offspring of GDM mothers, 2hPG was significantly higher (p = 0.0009) than in the controls (pooled MD: 0.43 mmol/L; 95% CI: 0.18–0.69; I^2^ = 35%; four studies, 890 children) (Table 2, S1C Fig).

#### Offspring of mothers with type 1 diabetes mellitus

**Obesity and overweight**

Two studies of offspring of T1DM mothers and controls were included in this review, involving a total of 577 children [26, 28].

**Fig 3:**
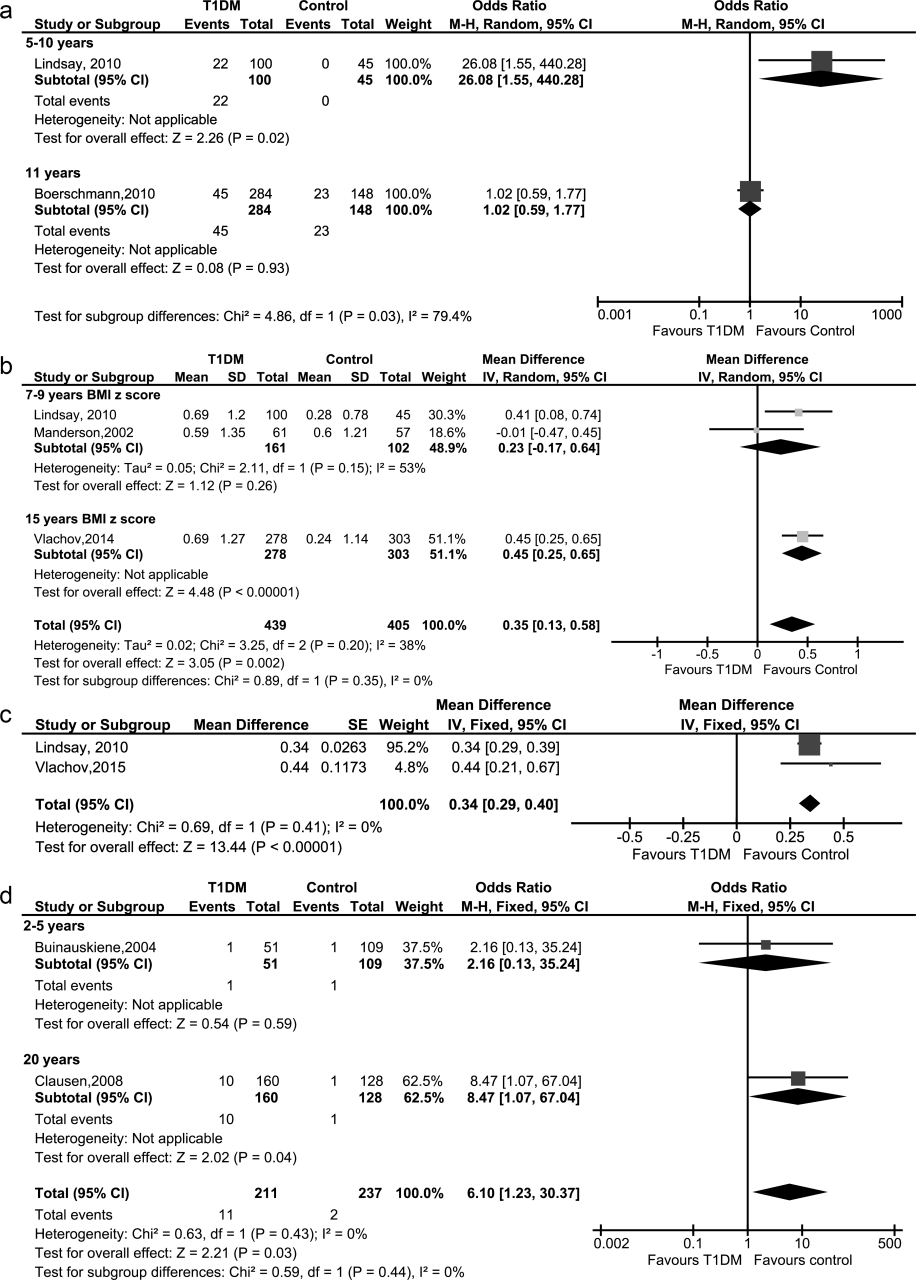
Forest plots of metabolic parameters in offspring of mothers with type 1 diabetes mellitus and controls: (a) obesity or overweight, (b) unadjusted BMI z-scores, (c) adjusted BMI z-scores, (d) diabetes.

No meta-analysis was conducted because of subgroup differences were detected. The rate of childhood obesity or overweight was significantly higher (p = 0.02) in the offspring of T1DM mothers among offspring aged 5 to 10 years old (OR: 26.08; 95% CI: 1.55–440.28; one study, 145 children), but there was no significant difference in 11 years old (OR: 1.02; 0.59–1.77; one study, 432 children) (Fig 3A). Quality of evidence was judged as ‘low’ for 5- to 10-year-olds and ‘very low’ among 11-year-olds (S2 Table).

**BMI z-scores**

Four studies of offspring of T1DM mothers and controls were included in this review, involving a total of 876 children [18, 19, 28, 30].

Unadjusted BMI z-scores of the offspring of T1DM mothers was significantly higher (p value = 0.002) than the controls (pooled MD: 0.35, 95% CI: 0.13–0.58, I^2^ = 38%, three studies, 844 children)(Fig 3B). Quality of the evidence was judged as ‘low’ (S2 Table). In a sensitivity analysis, overall unadjusted BMI z-scores of the offspring of T1DM mothers including Hunter et al. was significantly higher (p value = 0.002) than the controls (pooled MD: 0.45, 95% CI: 0.17–0.73, I^2^ = 64%, four studies, 876 children) (S3B Fig), we excluded Hunter et al. [18] because the domain of participant selection had high risk of bias [31] and reported as main results. Heterogeneity decreased from 64% to 38% but significance did not change after sensitivity analysis (S3B Fig).

Two studies [28, 30] were adjusted by maternal pre-pregnancy BMI. Adjusted BMI z-scores of the offspring of T1DM mothers were also significantly higher than those of the controls (pooled MD: 0.34; 95% CI: 0.29–0.40; two studies, 726 children) (Fig 3C).

**Diabetes**

Two studies of offspring of T1DM mothers and controls were included in this review, involving a total of 448 children [22, 25]. The rate of DM was significantly higher (p = 0.03) in the offspring of T1DM mothers. (pooled OR: 6.10; 95% CI: 1.23–30.37; I^2^ = 20%; two studies, 448 children) (Fig 3D). Quality of evidence was judged as ‘very low’ and was downgraded due to selective-outcome reporting (S2 Table).

From the viewpoint of abnormal glucose tolerance (total T2DM, IGT and IFG), a significantly (p<0.0001) higher rate of abnormal glucose tolerance was reported in offspring with T1DM (pooled OR: 3.48; 95% CI: 1.87–6.49; I^2 =^ 0%; two studies, 448 children) (Table 2, S1D Fig). Quality of evidence was judged as ‘low’ (S2 Table).

**Plasma glucose**

Five studies of offspring of T1DM mothers and controls were included in this review, involving a total of 1091 children for FPG and 915 children for 2hPG[18, 19, 22, 28, 30]. We excluded Hunter et al. [18]from the meta-analysis because the domain of participant selection was high risk.

For FPG, no meta-analysis was conducted because of subgroup differences were detected. No significant difference was found between T1DM and controls in 7- to 10-year-olds (pooled MD: -0.07 mmol/L; 95% CI:-0.16–0.03; I^2^ = 0%; two studies, 190 children), and in 20-year-olds (MD: 0.10 mmol/L; 95% CI:-0.00–0.20; 288 children). Significantly higher FPG was found in the offspring of T1DM mothers aged 15-year-olds (p value = 0.003, MD: 0.10 mmol/L; 95% CI: 0.03–0.17; 581 children) (Table 2, S1E Fig).

For 2hPG, no meta-analysis was conducted because of subgroup differences were detected. No significant difference was observed between the T1DM group and controls in 7- to 10-year-olds (MD: -0.60 mmol/L; 95% CI: -1.23–0.03; 46 children). However, significantly higher 2hPG was found in the offspring of T1DM mothers aged 15-year-olds (p value = 0.004, MD: 0.30 mmol/L; 95% CI: 0.10–0.50; 581 children) and 20-year-olds (MD: 0.50 mmol/L; 95% CI: 0.17–0.83; 288 children) (Table 2, S1F Fig).

#### Offspring of mothers with type 2 diabetes mellitus

**Offspring BMI z-scores, obesity and overweight**

We found only one study with BMI z-scores including the offspring of T2DM mothers [18]. Unadjusted BMI z-scores of the offspring of T2DM mothers aged 5–10 years old was significantly higher than the controls (MD: 3.40; 95% CI: 2.87–3.93; 25 children).

We found no studies for the rate of childhood obesity or overweight including the offspring of T2DM mothers.

**Diabetes, plasma glucose**

We found no studies with rates of childhood T2DM, including the offspring of T2DM mothers. Only one study was included for offspring FPG of mothers with T2DM and controls. FPG was significantly higher than in the controls (p value = 0.005, MD: 0.10 mmol/L; 95% CI: 0.03–0.17, 32 children)[18].

## Discussion

In this meta-analysis, we have shown that offspring of GDM mothers had a significantly higher risk of obesity or overweight in childhood than offspring of non-diabetic mothers. In addition, we found that offspring of GDM mothers had a higher 2hPG after glucose load from prepubertal to early adulthood compared with offspring of non-diabetic mothers. The offspring of T1DM mothers had significantly higher BMI z-scores from prepubertal to adolescent than offspring of non-diabetic mothers independently of maternal obesity. Also, the risk of diabetes or AGT was higher in those of T1DM mothers than in controls after the data of 2–5 year olds and 20 year olds were synthesized, while T1DM mothers generally do not have strong genetic background of T2DM. These findings suggested that childhood overweight/obesity or diabetes in offspring of T1DM mothers is likely due to the influence of intrauterine exposure to hyperglycemia.

In the studies on offspring of GDM mothers and controls (S2 Table), the evidence was judged to be of low quality (obesity and overweight, BMI z-score) and very low quality (DM, AGT), while in those on offspring of T1DM mothers and controls (S2 Table), the evidence was judged to be of low quality (BMI z-score, AGT) and very low quality (obesity and overweight, DM). These outcomes were downgraded due to observational studies bias limitation and wide 95%CI with small sample size.

Philipps et al. reported in their meta-analysis that unadjusted BMI z-scores of the offspring of GDM mothers were significantly higher than those of controls (pooled MD: 0.28; 95% CI: 0.05–0.51; six studies)[7], although it was no longer significant after adjustment for maternal pre-pregnancy BMI using three studies (two on GDM). Kim et al. reported that eight of 12 studies had significantly high crude ORs of childhood obesity (>95^th^ percentile) or overweight (>85^th^ percentile) for offspring of GDM mothers[6]. Kim et al. also mentioned only one study which was adjusted for covariates including maternal pre-pregnancy BMI and showed the association was no longer significant after adjustment [6, 27]. Since these two systematic reviews [6, 7] were published, 10 other studies have been reported, of which six [12, 14, 16, 17, 24, 30] were included in this review. We found that GDM mothers had a significantly higher BMI z-scores in childhood than offspring of non-diabetic mothers although the association was no longer significant after adjustment for maternal pre-pregnancy BMI, which supported previous reviews [6, 7]. On the other hand, a recent study showed that offspring of GDM mothers were significantly more likely to be overweight at an early age than those born to non-diabetic mothers [32]. Excessive weight gain during pregnancy is also related to large for gestational age at birth and childhood obesity [33]. Since there are many potential confounding factors for child overweight/obesity including maternal pre-pregnancy BMI, weight gain during pregnancy, duration or intensity of breastfeeding etc., further studies that adjust for those confounding factors are needed to clarify the association between intrauterine exposure to hyperglycemia and overweight/obesity in offspring of GDM mothers.

To detect mild glucose tolerance in the offspring of GDM mothers earlier, such as in school ages, it may be necessary to examine blood tests after glucose load. Regarding AGT among the offspring of GDM mothers, the influence of intrauterine exposure of hyperglycemia was suggested, although genetic factors of T2DM could not be distinguished because of high genetic predisposition to T2DM related to GDM.

We need to take into account the level of plasma glucose and the effects of therapeutic interventions on hyperglycemia of diabetic mothers, although in this systematic review information on maternal treatment during pregnancy was insufficient. Landon et al. showed that there was no apparent reduction in the rates of obesity or fasting glucose in treated offspring compared with control group at age 5 to 10 years, although the study showed only treated female children had lower fasting glucose [34]. We have shown that the offspring of T1DM mothers had significantly higher BMI z-scores from prepubertal to adolescent period compared with offspring of non-diabetic mothers.

We also showed that BMI z-scores of the offspring of T1DM mothers were significantly higher than those of controls after adjustment for maternal pre-pregnancy BMI. This means the intrauterine exposure of hyperglycemia would directly affect offspring higher BMI z-scores besides hereditary determinants. Hummel et al. reported that independent risk factor for childhood overweight in offspring of T1DM mothers was short breast-feeding duration and high birth weight, while maternal type 1 diabetes was not an independent predictor [35], which showed that intrauterine hyperglycemia might affect childhood overweight via birth size.

We have shown that the offspring of T1DM mothers had a higher risk of diabetes after the data of 2- to 5-year-olds and 20-year-olds were synthesized. Compared with the offspring of GDM mothers, the offspring of T1DM mothers seemed to be affected by metabolic effect from early age, regardless of low genetic predisposition to obesity or T2DM. One likely explanation is that offspring of women with T1DM are generally considered to be exposed to intrauterine hyperglycemia during the whole pregnancy period, while offspring of women with GDM are exposed to hyperglycemia only during the second half of the pregnancy. Early pregnancy period is thought to be a sensitive period in terms of metabolic effect on the offspring [36]. Another explanation is that offspring of T1DM mothers were exposed to a relatively high plasma glucose level compared with those of mothers with GDM. The possibility that the etiology of DM/ATG might be due to diminution of beta cells caused by pre-onset of T1 DM could not be denied. Sobngwi et al. found that offspring of mothers with T1DM had a higher occurrence of IGT compared with those of fathers with T1DM when they were around 20 years old, [37] suggesting that an exposure to hyperglycemia *in utero* is associated independent of the effect of genetic factors for T1DM. There were no reports about the effect of maternal diabetic control on offspring’s metabolic state after growth. The effect of the intervention into maternal blood glucose on offspring’s metabolic state should be assessed in the future.

### Limitations

This review has several limitations. First, the evidence of this review relies only on observational studies. Therefore, we could not confirm the causal relationship between intrauterine exposure to maternal hyperglycemia and offspring obesity or impaired glucose tolerance.

Second, most of the data included in this meta-analysis were crude data with no adjustment, because only a few studies in this review adequately adjusted for covariables [11, 12, 27, 28, 30]. Genetic, demographic, socioeconomic and lifestyle factors including postnatal growth and breastfeeding can influence obesity and diabetes, and should be adjusted for [38, 39].

## Conclusions

Exposure to maternal hyperglycemia during pregnancy might be associated with offspring obesity and abnormal glucose tolerance, although the association depends on the duration and intensity of intrauterine exposure to hyperglycemia, and the evidence relies only on observational studies with low quality of evidence. As for offspring of mothers with T2DM, we have to assess the actual status. To explore a causal relationship, well-designed prospective trials that consider the genetic background, the timing and strength of intrauterine exposure to hyperglycemia, other related factors and long-term results are warranted.

## Supporting information

S1 Fig Forest plots of metabolic parameters in offspring of mothers with gestational diabetes mellitus, type 1diabetes mellitus and controls: (a) abnormal glucose tolerance (GDM), (b) fasting plasma glucose (GDM), (c) 2h plasma glucose (GDM), (d) abnormal glucose tolerance (T1DM), (e) fasting plasma glucose (T1DM), (f) 2h plasma glucose (T1DM).
(TIF)

S2 Fig Risk of bias graph and risk of bias summary. (TIF)

S3 Fig Forest plots of sensitivity analysis: (a) BMI z-score (GDM), (b) BMI z-score (T1DM).
(TIF)

S1 Table Full search strategy. (DOCX)

S2 Table GRADE evidence profiles: Summary of findings comparing offspring with GDM mothers and controls and offspring with T1DM mothers and controls. (DOCX)

S3 Table PRISMA checklist. (DOC)

## Abbreviations

MD: mean difference
OR: odds ratio
T1DM: type 1 diabetes mellitus
T2DM: type 2 diabetes mellitus
GDM: gestational diabetes mellitus
IFG: impaired fasting glucose
IGT: impaired glucose tolerance
AGT: abnormal glucose tolerance
FPG: fasting plasma glucose
2hPG: two-hour plasma glucose
