# Supplementary figures and images for: Obesity and abnormal glucose tolerance in offspring of diabetic mothers: A systematic review and meta-analysis
Source: PLoS One. 2018 Jan 12;13(1):e0190676. doi: 10.1371/journal.pone.0190676 (PMC5766126; doi:10.1371/journal.pone.0190676)

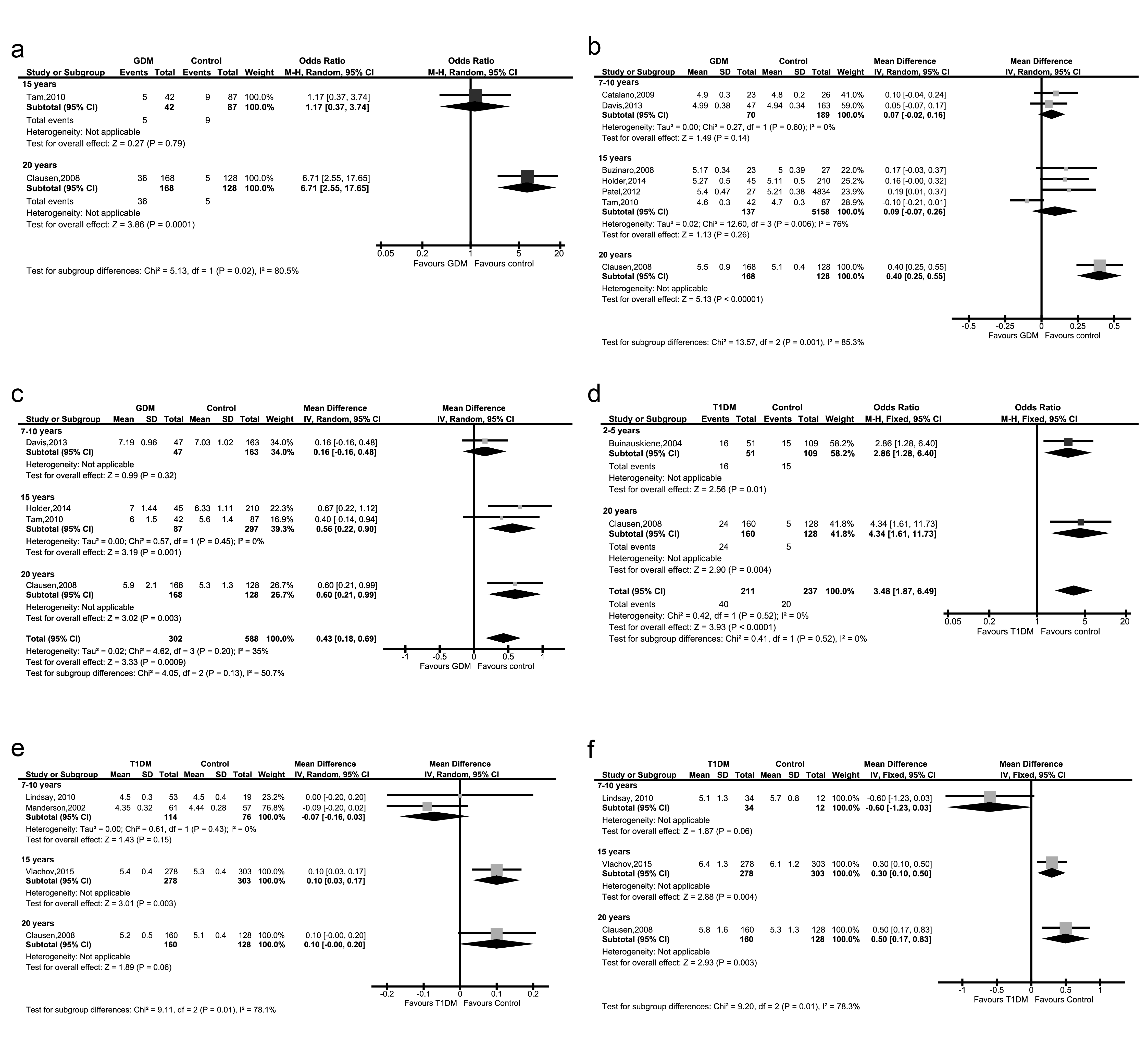

Supplement: S1 Fig — Forest plots of metabolic parameters in offspring of mothers with gestational diabetes mellitus, type 1diabetes mellitus and controls: (a) abnormal glucose tolerance (GDM), (b) fasting plasma glucose (GDM), (c) 2h plasma glucose (GDM), (d) abnormal glucose tolerance (T1DM), (e) fasting plasma glucose (T1DM), (f) 2h plasma glucose (T1DM). (TIF) [file pone.0190676.s001.tif]

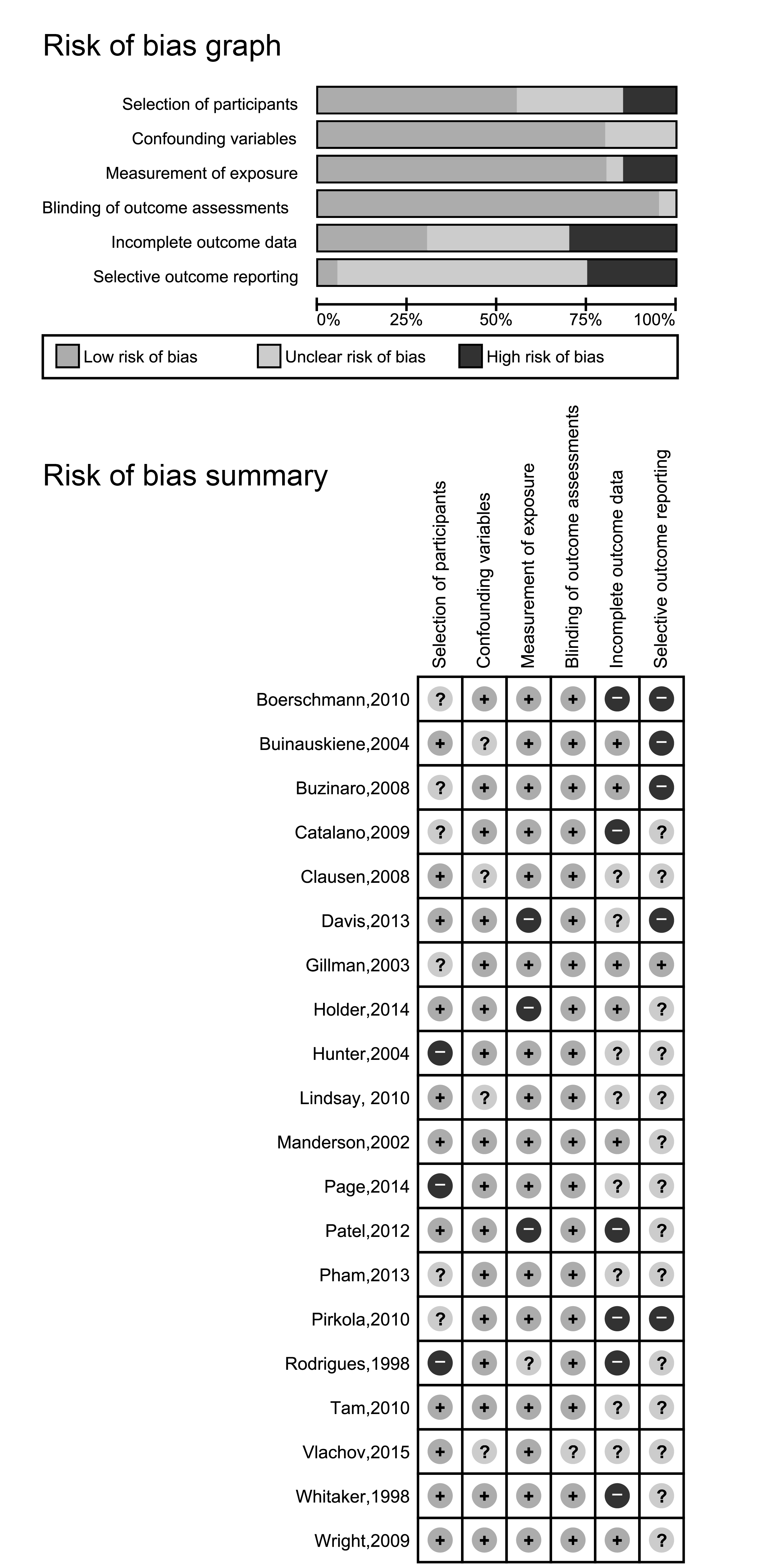

Supplement: S2 Fig — (TIF) [file pone.0190676.s002.tif]

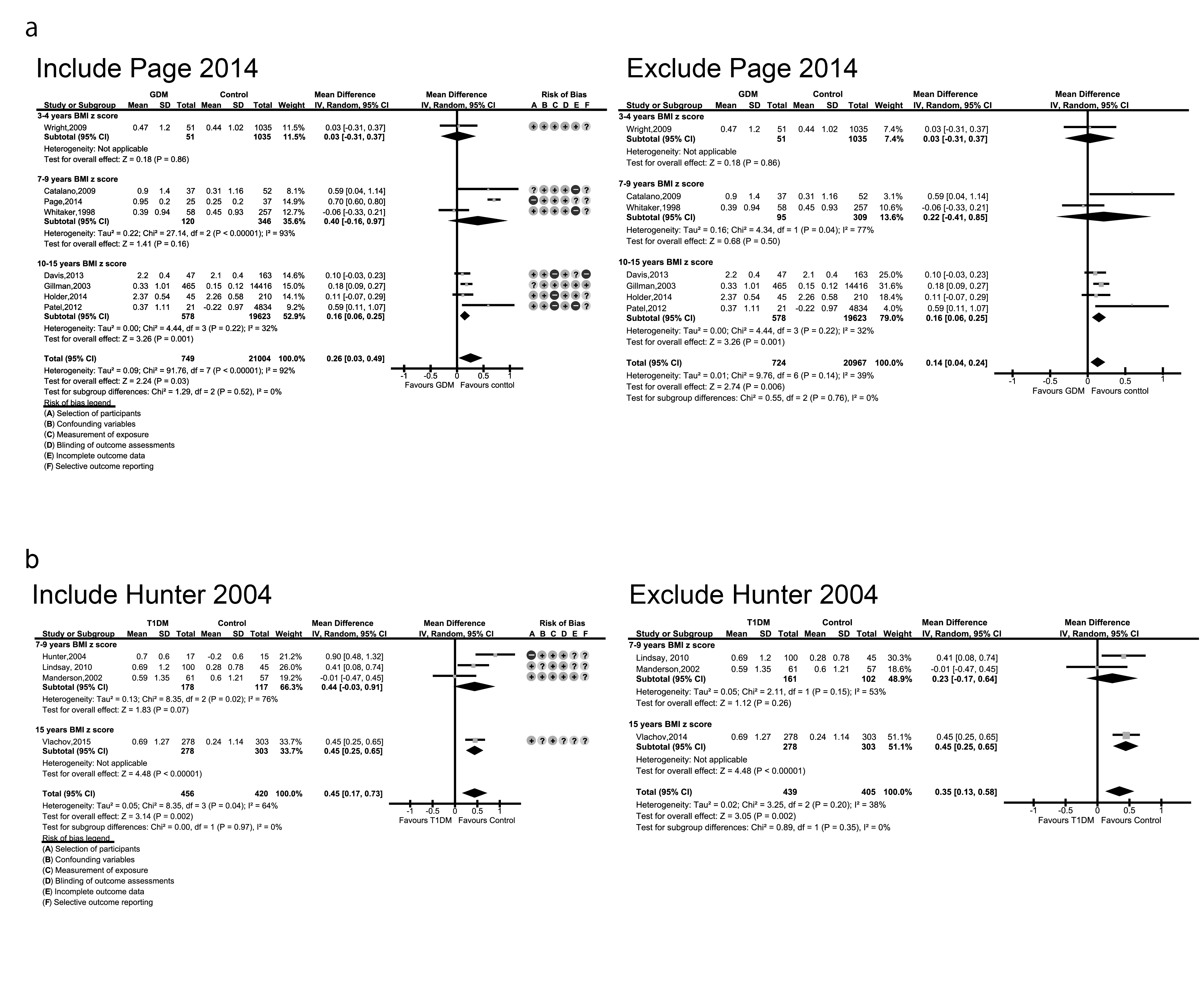

Supplement: S3 Fig — Forest plots of sensitivity analysis: (a) BMI z-score (GDM), (b) BMI z-score (T1DM). (TIF) [file pone.0190676.s003.tif]
