# Supplementary material for: Obesity and abnormal glucose tolerance in offspring of diabetic mothers: A systematic review and meta-analysis
Source: PLoS One. 2018 Jan 12;13(1):e0190676. doi: 10.1371/journal.pone.0190676 (PMC5766126; doi:10.1371/journal.pone.0190676)
Supplement: S1 Table — (DOCX) [file pone.0190676.s004.docx]

Supplementary Table1. Full search strategy

**MEDLINE (October 03, 2014)**

Database:

Ovid MEDLINE(R) In-Process & Other Non-Indexed Citations <October 03, 2014>,

Ovid MEDLINE(R) 1946 to Present with Daily Update

--------------------------------------------------------------------------------

1 exp *Diabetes Mellitus/ (255957)

2 Pregnancy in Diabetics/ (9894)

3 Diabetes, Gestational/ (6032)

4 (diabetic adj (mother* or parent* or pregnanc* or pregnant or wom?n)).tw. (6726)

5 or/1-4 (263948)

6 "Child of Impaired Parents"/ (4164)

7 Maternal Exposure/ (5532)

8 Prenatal Exposure Delayed Effects/ (21019)

9 ((exposure or exposed) adj10 (prenatal* or antenatal* or utero or intrauterine or fetal or maternal)).tw. (25995)

10 or/6-9 (44388)

11 5 and 10 (730)

12 (diabetes or diabetic or dm).tw. (437841)

13 (impaired adj (mother* or parent* or pregnanc* or pregnant)).tw. (118)

14 or/4,9,13 (32731)

15 12 and 14 (7305)

16 15 not medline.st. (332)

17 or/11,16 (1055)

18 exp animals/ not humans.sh. (4019741)

19 17 not 18 (919)

20 remove duplicates from 19 (879)

***************************

**MEDLINE update (September 25, 2015)**

Database:

Ovid MEDLINE(R) In-Process & Other Non-Indexed Citations <September 25, 2015>,

Ovid MEDLINE(R) 1946 to Present with Daily Update

--------------------------------------------------------------------------------

1 exp *Diabetes Mellitus/ (270598)

2 Pregnancy in Diabetics/ (10089)

3 Diabetes, Gestational/ (6697)

4 (diabetic adj (mother* or parent* or pregnanc* or pregnant or wom?n)).tw. (6948)

5 or/1-4 (278804)

6 "Child of Impaired Parents"/ (4371)

7 Maternal Exposure/ (6106)

8 Prenatal Exposure Delayed Effects/ (22457)

9 ((exposure or exposed) adj10 (prenatal* or antenatal* or utero or intrauterine or fetal or maternal)).tw. (28082)

10 or/6-9 (47627)

11 5 and 10 (835)

12 (diabetes or diabetic or dm).tw. (470713)

13 (impaired adj (mother* or parent* or pregnanc* or pregnant)).tw. (128)

14 or/4,9,13 (35038)

15 12 and 14 (7613)

16 15 not medline.st. (412)

17 or/11,16 (1239)

18 exp animals/ not humans.sh. (4116467)

19 17 not 18 (1088)

20 remove duplicates from 19 (1018)

21 limit 20 to ed="20140901 - 20150928" (114)

***************************

**EMBASE (October 03, 2014)**

Platform: Embase.com

| No. | Query | Results |
| --- | --- | --- |
| #11 | #8 OR #10 | 1,444 |
| #10 | #3 AND #9 | 1,217 |
| #9 | 'progeny'/de AND [embase]/lim NOT [medline]/lim | 12,684 |
| #8 | #3 AND #7 | 693 |
| #7 | #4 OR #5 OR #6 | 21,721 |
| #6 | ((exposure OR exposed) NEAR/10 (prenatal OR antenatal OR utero OR intrauterine OR fetal OR maternal)):ab,ti AND [embase]/lim NOT [medline]/lim | 7,684 |
| #5 | 'environmental exposure'/de AND [embase]/lim NOT [medline]/lim | 13,898 |
| #4 | 'prenatal exposure'/de AND [embase]/lim NOT [medline]/lim | 2,729 |
| #3 | #1 OR #2 | 229,784 |
| #2 | (diabetic NEAR/1 (mother OR mothers OR parent OR parents OR pregnancy OR pregnancies OR pregnant OR woman OR women)):ab,ti AND [embase]/lim NOT [medline]/lim | 2,303 |
| #1 | 'diabetes mellitus'/exp AND [embase]/lim NOT [medline]/lim | 229,642 |

**EMBASE update (February 12, 2016)**

Platform: Embase.com

| #13 | #11 NOT #12 | 471 |
| --- | --- | --- |
| #12 | 'animal'/exp NOT 'human'/de AND [embase]/lim NOT [medline]/lim AND [1-9-2014]/sd NOT [28-9-2015]/sd | 101,665 |
| #11 | #8 OR #10 | 554 |
| #10 | #3 AND #9 | 358 |
| #9 | 'progeny'/de AND [embase]/lim NOT [medline]/lim AND [1-9-2014]/sd NOT [28-9-2015]/sd | 3,752 |
| #8 | #3 AND #7 | 248 |
| #7 | #4 OR #5 OR #6 | 5,525 |
| #6 | ((exposure OR exposed) NEAR/10 (prenatal OR antenatal OR utero OR intrauterine OR fetal OR maternal)):ab,ti AND [embase]/lim NOT [medline]/lim AND [1-9-2014]/sd NOT [28-9-2015]/sd | 2,306 |
| #5 | 'environmental exposure'/de AND [embase]/lim NOT [medline]/lim AND [1-9-2014]/sd NOT [28-9-2015]/sd | 3,307 |
| #4 | prenatal exposure'/de AND [embase]/lim NOT [medline]/lim AND [1-9-2014]/sd NOT [28-9-2015]/sd | 827 |
| #3 | #1 OR #2 | 51299 |
| #2 | (diabetic NEAR/1 (mother OR mothers OR parent OR parents OR pregnancy OR pregnancies OR pregnant OR woman OR women)):ab,ti AND [embase]/lim NOT [medline]/lim AND [1-9-2014]/sd NOT [28-9-2015]/sd | 336 |
| #1 | 'diabetes mellitus'/exp OR 'diabetes mellitus' AND [embase]/lim NOT [medline]/lim AND [1-9-2014]/sd NOT [28-9-2015]/sd | 51,285 |
