# Supplementary material for: Obesity and abnormal glucose tolerance in offspring of diabetic mothers: A systematic review and meta-analysis
Source: PLoS One. 2018 Jan 12;13(1):e0190676. doi: 10.1371/journal.pone.0190676 (PMC5766126; doi:10.1371/journal.pone.0190676)
Supplement: S2 Table — (DOCX) [file pone.0190676.s005.docx]

Supplementary Table 2. GRADE evidence profiles: Summary of findings comparing offspring with GDM mothers and controls and offspring with T1DM mothers and controls

| **Obesity·and·diabetes·in··offspring·compared·with·offspring·of·GDM·mothers·and·controls¤** | | | | | | |
| --- | --- | --- | --- | --- | --- | --- |
| **Patient·or·population**: offspring·of·GDM·mothers·and·controls¶  **Exposure**: offspring·of·GDM·mothers¶  **Comparison**: offspring·of·non-diabetic·mothers¤ | | | | | | |
| **Outcomes¤** | **Anticipate· absolute·effects^*^ (95% CI) ¶** | | **Relative· effect¶ (95%· CI) ¤** | **№ of· participants¶ (studies) ¤** | **Quality·of· the· evidence (GRADE) ¤** | **Comments¤** |
|  | **Risk·with·control·mothers¤** | **Risk·with·GDM mothers¤** |  |  |  |  |
| **Obesity·or·overweight¶**  **2-17·years¤** | **Study·population ¶** | | **OR· 1.35 ¶** (1.01·to·1.80)¤ | 19559¶ (8· observationa·l studies)¤ | ⨁⨁◯◯¶ LOW¤ | ¤ |
|  | 97 per 1,000¶ | **126·per· 1,000·** (98· to ·162) ¶ |  |  |  |  |
|  | **Moderate¶** | |  |  |  |  |
|  | 167· per ·1,000¤ | **213·per· 1,000**· (168· to ·265)¤ |  |  |  |  |
| **BMI·z-score¶**  **3-15·years¤** | The· mean· BMI· z-score· was· **0¤** | The·mean· BMI· z-score· of· the· offspring ·of ·GDM· mothers· was· **0.14· higher·** (0.04 ·higher· to· 0.24· higher)· than· the· controls.¤ | - | 21691¶ (7·observational·studies)¤ | ⨁⨁◯◯¶ LOW¤ | ¤ |
| **Diabetes¶**  **15-·20 years¤** | 5· per·1,000¤ | **26· per· 1,000**· (4· to· 137) ¤ | **OR 5.70¶** (0.96· to· 33.97¤) | 425¶ (2· observational· studies)¤ | ⨁◯◯◯¶ VERY LOW^1,2^¤ | ¤ |
| **Abnormal·glucose·tolerance¶**  **15·years**¶ | 103· per· 1,000¤ | **119· per ·1,000·** (41· to ·301) ¶ | **OR 1.17¶** (0.37· to· 3.74) ¶ | 129¶ (1· observational· study) ¤ | ⨁◯◯◯¶ VERY LOW^1,2^¤ | ¶ |
| **20·years ¤** | 39· per·1,000¤ | **214· per ·1,000** · (94· to ·418) ¤ | **OR 6.71¶** (2.55· to· 17.65) ¤ | 296¶ (1· observational· study) ¤ | ⨁◯◯◯¶ VERY LOW^1,2^¤ | ¤ |
| **Obesity·and·diabetes·in·offspring·compared· with·offspring·of·T1DM·mothers·and·controls** | | | | | | |
| **Patient· or· population**: offspring· of· T1DM· mothers· and· controls¶  **Exposure**: offspring· of ·T1DM ·mothers ¶  **Comparison**: offspring· of· non-diabetic· mothers ¤ | | | | | | |
| **Outcomes¤** | **Anticipate· absolute·effects^*^ (95% CI) ¶** | | **Relative· effect¶ (95%· CI) ¤** | **№· of· participants¶  (studies) ¤** | **Quality· of· the ·evidence¶ (GRADE) ¤** | **Comments¤** |
|  | **Risk· with· contro·l mothers¤** | **Risk· with· T1DM ·mothers¤** |  |  |  |  |
| **Obesity·or·overweight ¶**  **5-10· years ¶** | 0 ·per· 1,000 ¶ | **0· per· 1,000·** (0· to· 0) ¶ | **OR·26.08¶** (1.55 ·to· 440.28) ¤ | 145¶ (1· observationa·l study¤) | ⨁◯◯◯¶ VERY LOW ^2^¤ | ¶ |
| **≧11·years¤** | **Study·population¶** | | **OR 1.02** ¶  (0.59· to ·1.77)¤ | 432 ¶  (1· observationa·l study)¤ | ⨁◯◯◯¶ VERY LOW ^2^¤ | ¤ |
|  | 155· per· 1,000¶ | **158· per·1,000·** (98· to· 246)¶ |  |  |  |  |
|  | **Moderate¶** | |  |  |  |  |
|  | 155· per ·1,000¤ | **158· per ·1,000·** (98· to· 246)¤ |  |  |  |  |
| **BMI· z-score**¶  **7-15·years¤** | The ·mean· BMI· z-score·　　T1DM ·was· **0** ¤ | The· mean· BMI· z-score· of· the ·offspring· of ·T1DM· mothers· was· **0.35· higher** (0.13 ·higher· to· 0.58· higher)· the· controls.¤ | - | 844¶ (3 ·observationa·l studies) ¤ | ⨁⨁◯◯¶ LOW ¤ | ¤ |
| **Diabetes¶**  **2-5·years,20· years¤** | **Study population¶** |  | **OR· 6.10¶** (1.23· to ·30.37) ¤ | 448¶ (2·observationa·studies) ¤ | ⨁◯◯◯¶ VERY LOW ^2^¤ | ¤ |
|  | 8· per 1,000 ¶ | **49· per· 1,000·** (10· to· 205) ¶ |  |  |  |  |
|  | **Moderate¶** |  |  |  |  |  |
|  | 9· per·1,000 ¤ | **50· per· 1,000·** (1· to· 246) ¤ |  |  |  |  |
| **Abnormal· glucose· tolerance¶**  **2-5· years,20 ·years¤** | **Study population¶** | | **OR· 3.48** ¶ (1.87· to·6.49) ¤ | 448¶ (2· observational· studies) ¤ | ⨁⨁◯◯¶ LOW¤ | ¤ |
|  | 84· per· 1,000 ¶ | **243· per· 1,000·** (147· to· 374)¶ |  |  |  |  |
|  | **Moderate¶** |  |  |  |  |  |
|  | 88· per· 1,000 ¤ | **252· per· 1,000·** (147 ·to ·374)¤ |  |  |  |  |
| ***The· risk· in· the· intervention· group** (and· its· 95%· confidence· interval) is· based ·on· the· assumed· risk· in· the· comparison·group·and· the· **relative· effect**·of· the· intervention· (and· its· 95%· CI). ¶ **CI:** Confidence· interval; **MD:** Mean· difference; **RR:** Risk· ratio; **OR:** Odds· ratio ¤ | | | | | | |

1. High dropout rate.
2. Wide 95%CI and small sample size.
